# Simultaneous Quantification of Antioxidant Compounds in *Phellinus igniarius* Using Ultra Performance Liquid Chromatography-Photodiode Array Detection-Electrospray Ionization Tandem Mass Spectrometry

**DOI:** 10.1371/journal.pone.0163797

**Published:** 2016-09-30

**Authors:** Dan Shou, Yu Dong, Nani Wang, Hongyu Li, Yang Zhang, Yan Zhu

**Affiliations:** 1 Department of Chemistry, Zhejiang University, Tianmushan Road 148, Hangzhou 310028, China; 2 Department of Medicine, Zhejiang Academy of Traditional Chinese Medicine, Tianmushan Road 132, Hangzhou 310007, China; University of Edinburgh, UNITED KINGDOM

## Abstract

Natural antioxidants are widely used in the life sciences. *Phellinus igniarius* is a historically used natural antioxidant containing a variety of active compounds. Phenols, particularly Inoscavin A and Hypholomine B, are found in the high concentrations. Better quantitative methods are needed to perform quality control in order to support further research of this mushroom. An ultra-performance liquid chromatography method coupled to photodiode-array detection and an electrospray ionization tandem mass spectrometry method (UPLC-PAD-MS) was developed to simultaneously quantify Inoscavin A and Hypholomine B levels in the medicinal fungus *Phellinus igniarius*. The two compounds were quantified using UPLC-PAD and UPLC-MS. The methods were accurate (mean accuracy for spiked matrix ranged from 101.5% to 105.8%), sensitive (limit of detection ranged from 0.28 to 1.14 mg L^-1^) and precise (the relative standard deviations ranged from 0.13 to 2.8%). Inoscavin A and Hypholomine B were purified using high-speed counter-current chromatography (HSCCC), structural evaluated to meet the request of standard substances. UPLC separation was performed on a reversed-phase C18 column using gradient elution with acetonitrile and 0.1% formic acid over 10 min. The developed method was successfully applied to determine Inoscavin A and Hypholomine B in twelve *Phellinus igniarius* samples of different origins and the results showed that it was suitable for the analysis of these active components in *Phellinus igniarius* samples.

## Introduction

**Fig 1:**
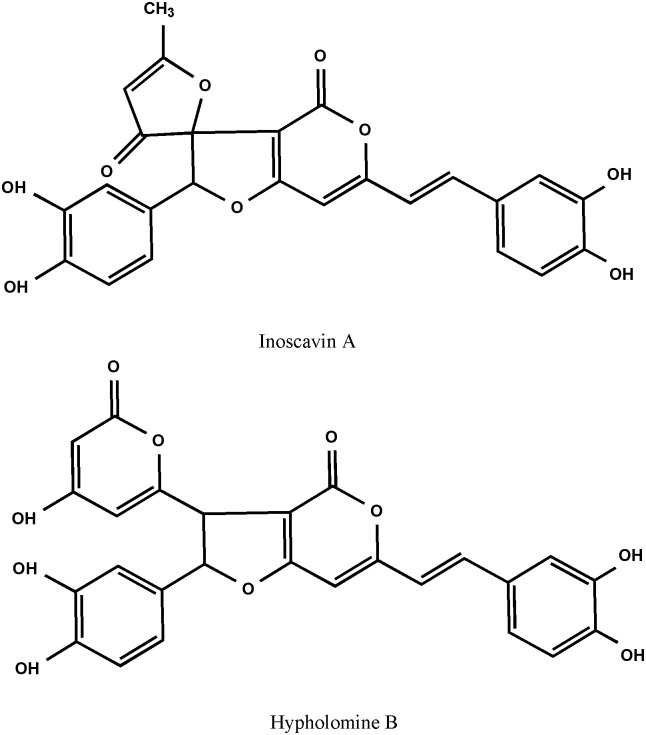
The structure of Inoscavin A and Hypholomine B.

Mushroom is an important source of biologically active compounds with medicinal value [1]. *Phellinus igniarius* is a kind of mushroom historically used for food, and in folk medicine, it was also used for the treatment of a variety of human diseases, such as cancer, influenza and heart diseases. No adverse effects have been reported about its use [2, 3]. A variety of secondary metabolites, including phenolic compounds, polyketides, terpenes, steroids and polysaccharides have been identified. The polyphenols from *Phellinus igniarius* attract major interest because of their potential anticarcinogenic properties [4, 5], presumably based on their function as natural antioxidants [6]. Inoscavin A and Hypholomine B (Fig 1) are of particular interest because they are found in high concentrations in *Phellinus igniarius* [7]. Better quantitative methods are needed to perform quality control in order to support further research of this mushroom.

A method for the simultaneous separation and quantification of Inoscavin A and Hypholomine B has not been described due to a lack of standards. High-speed counter-current chromatography (HSCCC) is a support-free liquid-liquid partition chromatography technique that is widely used for the purification of natural products [8, 9], including the purification of polyphenols from natural sources [10,11]. The structure of these two polyphenols has been carefully evaluated using nuclear magnetic resonance spectroscopy (NMR), mass spectrometry (MS) [12,13] and ultraviolet spectrometry (UV) [14]. The data was contained in supporting information and proved being in accordance with the previously researching report [12, 15].

The separation of natural compounds, such as polyphenols is usually carried out by using high-performance liquid chromatography (HPLC) methods [16,17]. The complex mixture of components in plant extracts has led to long analysis times, poor resolution and large solvent consumption with HPLC. Ultra performance liquid chromatography (UPLC) has been applied to study polyphenols in plant materials with better success [18]. UPLC separation columns use a new column technology which has allowed the UPLC system to run routinely at the pressures up to 15,000 psi. Therefore, the UPLC system had a higher sensitivity, higher resolution and a shorter analysis time than HPLC.

In this paper, a rapid and sensitive method was established for the simultaneous quantification of Inoscavin A and Hypholomine B in *Phellinus igniarius* samples, and thepreparation of standards for analysis with HSCCC was also described. What’s more, an UPLC-photodiode array detection-mass spectrometry method (UPLC-PAD-MS) was developed to separate and quantify target compounds found in *Phellinus igniarius*. Finally, we used twelve real samples to verify the developed method and demonstrate that the UPLC-PAD-MS method might be a useful alternative to the quality assessment for *Phellinus igniarius*.

## Experimental

### Reagents and chemicals

Acetonitrile (ACN, chromatography grade) was purchased from Tesia (Fairfield, USA). Formic acid (chromatography grade) was purchased from ROE (Newark, USA). Methylene chloride (analytical reagent grade) was obtained from the Shanghai Chemical Reagent Company (Shanghai, China). Deionized water (16 MΩ cm^-1^) was generated in-house using a Millipore Milli-Q Plus system (Bedford, MA, USA). *Phellinus igniarius* for isolation of Inoscavin and Hypholomine was purchased from Zhejiang Qingzheng Biotechnology Co. Ltd., Hangzhou, China.

### Sample preparation

First of all, the dried sporocarp of *Phellinus igniarius* were ground to powder (about 100 mesh) with a plant disintegrator (FW177, Taisite, Tianjin, China). Secondly, the powder (0.25 g) was extracted in 20 mL ethanol with sonication for 30 min. Finally, the extracts were filtered and then stored in the refrigerator for subsequent UPLC separation.

### Standard preparation

Inoscavin A and Hypholomine B standards were prepared with HSCCC. The HSCCC instrument used in this study was the TBE-300B HSCCC (Tauto Biotech, Shanghai, China) with multilayer coils (diameter of tube, 1.6 mm, total volume 230 mL). HSCCC separations were performed at a revolution speed of 800 rpm at 25°C. The coil column was first filled with the stationary phase. The apparatus was then rotated at 800 rpm while the low phase was pumped into the column at a flow rate of 1.5 mL min^-1^. After the system established hydrodynamic equilibrium, 10 mL sample solution containing 0.5 g ethanol extract of *Phellinus igniarius* was injected. The eluent from the column was collected in a Spectra/Chrom (USA) CF-1 collector (10 min per tube). The combined target fraction was evaporated under reduced pressure at 35°C and analyzed by UPLC-HDMS, UV, and NMR. The purities of the target compounds were verified to be over 95%. The data was contained in supporting information which was then used as the standard substances.

### UPLC-PAD-MS system

UPLC was performed using the Acquity ultra-high-performance liquid chromatography-tandem-quadrupole detection mass spectrometry (UPLC-TQD-MS) system (Waters, Milford, MA, USA), including a binary solvent delivery system, an autosampler, a photodiode array detector (PAD), and a mass spectrometry (MS). Analyses were performed by an Acquity UPLC BEH C_18_ column. The mobile phase consisted of (A) ACN containing 0.1% formic acid and (B) 0.1% formic acid in water. The UPLC separation conditions were optimized by a linear gradient from 20% to 30% B (0–8 min) and isocratic gradient at 30% A (8–10 min) with a flow rate of 0.3 mL min^-1^. The column and auto-sampler were maintained at 35°C and 25°C, respectively. The online PAD detector was recorded in the range of 190–400 nm, and the maximum absorption wavelength was 395 nm. The sample volume was 1 μL.

The ESI source was set in the negative ion mode. All quantifications were performed using the multiple reaction monitoring (MRM) mode. For MRM data collection during the UPLC experiments, the capillary voltage was 2.8 KV, the source temperature was 120°C, the desolvation temperature was 350°C, the desolvation gas flow was 600 L h^-1^, and the cone gas flow was 60 L h^-1^. The MRM transitions for the target compounds were 461 > 377 for Inoscavin A and 489 > 445 for Hypholomine B. The collision gas was argon and the flow rate was 0.14 mL/min. The collision voltage of hypholomine B and inoscavin A was 10 eV and 20 eV, respectively.

### HPLC-UV system

HPLC analysis was performed on a SHISEIDO LC-20A HPLC system with an auto sampler and a diode array detector. The analysis was conducted using an SHISEIDO CAPCELL PAK C_18_. UV absorption was measured at 395 nm. The mobile phase consisted of (A) methanol and (B) 0.2% phosphoric acid in water. The gradient program used a linear gradient from 30% to 40% A (0–20 min) and from 40% to 45% A (20–30 min), an isocratic gradient at 45% A (30–45 min) and a linear gradient from 45% to 55% A (45–60 min). The flow rate was kept constant at 1.0 mL min^-1^. The column temperature was 30°C. The wavelength was 395 nm. The sample injection volume was 10 μL.

## Results and Discussion

### Preparation of standard compounds

The Inoscavin A and Hypholomine B standards were prepared by the HSCCC method. In a HSCCC separation, the selection of the two-phase solvent system is the first and most critical step. The K values were measured by HPLC as previously reported [19]. For an efficient separation, the partition coefficient (K) values of the target compound should be close to 1 [20]. A larger K value tends to give broader peaks, while a smaller K value might give low resolution. In this work, the solvent system composed of petroleum ether-ethyl acetate-methanol-water was selected according to the polarity of the target compounds [21]. The K values for the target compounds of several two phase solvent systems are shown in Table 1. A solvent system composed of petroleum ether-ethyl acetate-methanol-water (1:0.9:1:1.5, by volume) was used to isolate Inoscavin A. Petroleum ether-ethyl acetate-methanol-water (1:1.7:1:2, by volume) was used as a solvent to isolate Hypholomine B. The purity of the two compounds was over 95%, as determined by the HPLC and HDMS.

**Table 1:** The K values of the target compounds in the two-phase solvent system composed of petroleum ether-ethyl acetate-methanol-water.

Solvent system (v/v)	Inoscavin A	Hypholomine B
0.5:2:1:2.5	0.08	3.10
0.8:2:1:2	0.10	1.12
0.9:1.5:1:2	0.31	1.59
1:0.9:1:1.5	0.93	0.02
1:1.7:1:2	0.15	1.07
1:2:1:2.5	0.26	1.66

In this section, the samples were directly extracted by ethanol and had a simple pre-treatment method. And we also used a same mixed standard solution as the quantity control sample in the sample testing process to monitor equipment stability. However a known substance with known quantity was not added as an internal standard for variations in extraction efficiencies and that may be a limitation of this study.

### Optimization of separation conditions in the UPLC system

**Fig 2:**
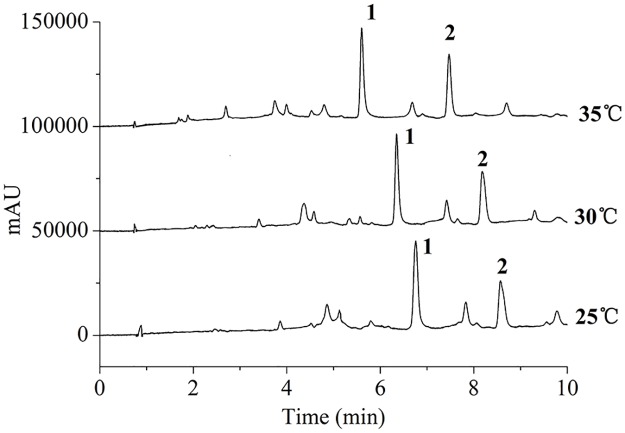
The effects of column temperature. The separation conditions were the mobile phase consisted of (A) 0.1% formic acid in water and (B) ACN containing 0.1% formic acid, linear gradient from 20% to 30% B (0–8 min), and isocratic at 30% B (8–10 min), at a flow rate of 0.3 mL min^-1^.

When using methanol as the organic phase, the chromatographic peaks had low resolution, low efficiency and long retention times. The separation was significantly improved using ACN as the organic phase. 0.1% formic acid was used to minimize peak tailing. The temperature of the column was another important variable to be taken into account. Fig 2 presented the effects of column temperature. It was found that a higher temperature produced a shorter retention time [21]. Moreover, the column temperature had no significant effect on the peak areas of the target compounds. Therefore, 35°C was selected as the column temperature.

**Fig 3:**
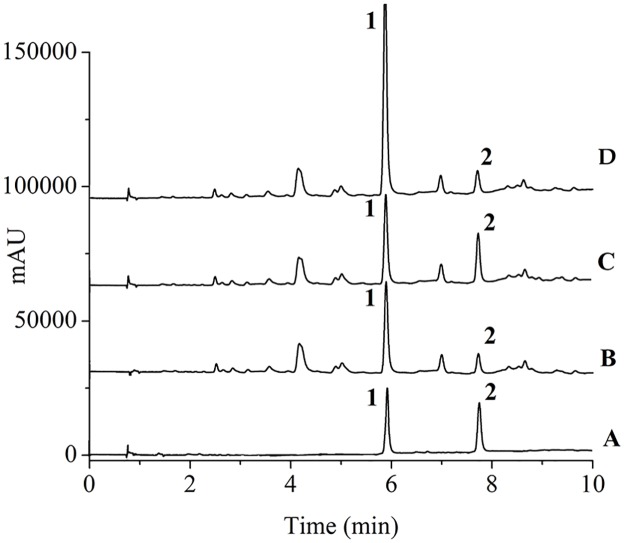
UPLC-PAD chromatograms of (A) Standard; (B) blank sample; (C) sample spiked with Inoscavin A (20 mg L^-1^); (D) sample spiked with Hypholomine B (90 mg L^-1^). Hypholomine B (1), Inoscavin A (2). The separation conditions were the mobile phase consisted of (A) 0.1% formic acid in water and (B) ACN containing 0.1% formic acid, linear gradient from 20% to 30% B (0–8 min), and isocratic at 30% B (8–10 min), at a flow rate of 0.3 mL min^-1^.

A PAD detector was suitable for the quantification of the polyphenols. It had a high sensitivity level for the polyunsaturated species [21]. 395 nm was chosen as the detection wavelength, as it was close to the maximum absorbency of the target compounds. Typical UPLC-PAD chromatograms are shown in Fig 3. Under optimized conditions, the target compounds produced well-defined, well-separated peaks at retention times of 5.8 min for Hypholomine B and 7.6 min for Inoscavin A.

**Fig 4:**
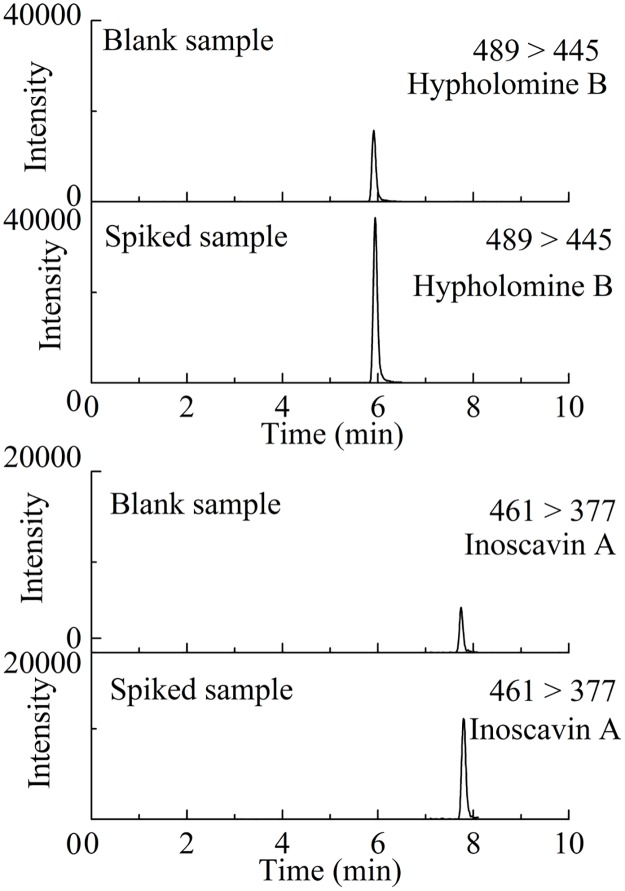
MRM chromatograms of (A) blank sample and sample spiked with Inoscavin A (20 mg L^-1^); (B) blank sample and sample spiked with Hypholomine B (90 mg L^-1^). Hypholomine B (1), Inoscavin A (2).

Moreover, the quantification of the target compounds could also be achieved by MS detection. More detailed structural information can be obtained when a MS detector was coupled with a PAD detector [22]. These compounds were well ionized in the negative ion mode. Hypholomine B and Inoscavin A were at first characterized by MS [23] scan and MS/MS product ions to ascertain their parent ions and to select product ions for use in MRM mode, respectively. To get the richest relative abundance of parent ions and product ions, the parameters for fragmentor energies and collision energies were optimized, and the MRM transitions were chosen to be *m/z* 489–445 for Hypholomine B and *m/z* 461–377 for Inoscavin A. A typical data collection mode for quantification by tandem quadrupole MS was MRM. Typical MRM chromatograms of target compounds are shown in Fig 4.

### Method validation

All calibration curve parameters are summarized in Table 2. Limits of detection (LODs) of the target compounds with both detectors were calculated as the amount corresponding to three times the noise recorded in chromatograms (S/N = 3). The resulting data (Table 2) demonstrated the higher sensitivity of MS detection than the PAD. Inoscavin A and Hypholomine B are styryl pyrone with four phenolic hydroxyl, which have the high ionization efficiency. This is the reason why the sensitivity of MS was higher than that of PAD [24]. The TQD mass spectrometry has the advantage in quantitative analysis due to its MRM mode, which can eliminate background interference [25].

**Table 2:** Calibration results and the LODs under optimal chromatographic conditions, as described in section 2.4.

Parameter	Inoscavin A		Hypholomine B	
	PAD	MS	PAD	MS
Linearity range (mg L^-1^)	2.7–400	1.3–400	3.8–300	0.93–300
Correlation, r	1	0.9997	0.9991	0.9994
Slope	40808	27282	42997	21408
Intercept	-51.823	-31.306	-84.093	-34.524
LODs (mgL^-1^)	0.81	0.39	1.14	0.28

Intra- and inter-day variations were chosen to determine the precision of the method. For inter-day variability determination, the two standard solutions (1.7 mg L^-1^ Inoscavin A and 1.8 mg L^-1^ Hypholomine B) were examined in duplicate for 3 consecutive days. For intra-day variability determination, 6 replicate solutions were analyzed within 24 hours. Variations were expressed as the relative standard deviation (R.S.D.). Table 3 shows that there was little variation of retention time and peak.

**Table 3:** Repeatability of retention time and peak area.

Target compound	Inter-repeatability		Intra-repeatability	
	(R.S.D., %, n = 6)		(R.S.D., %, n = 3)	
	Retention time	Peak area	Retention time	Peak area
Inoscavin A _PAD_	0.13	2.6	0.17	2.8
Hypholomine B _PAD_	0.26	1.2	0.24	1.3
Inoscavin A _MS_	0.15	1.6	0.19	1.7
Hypholomine B _MS_	0.23	0.92	0.25	1.3

The accuracy of the proposed method was tested using recovery measurements of spiked samples. The results are summarized in Table 4. The recoveries were within the range of 101.5–105.8%.

**Table 4:** Recoveries of Inoscavin A and Hypholomine B from *Phellinus igniarius* sample (n = 3).

Target compound	Content (mg)	Spiked amount (mg)	Found amount (mg)	Recovery (%)
Inoscavin A _PAD_	0.0474	0.0237	0.0720	103.9
		0.0474	0.0962	103.0
		0.0711	0.1211	103.6
Hypholomine B _PAD_	0.0688	0.0344	0.1037	101.5
		0.0688	0.1399	103.3
		0.1032	0.1752	103.1
Inoscavin A _MS_	0.0525	0.0237	0.0771	103.8
		0.0525	0.1026	105.8
		0.0711	0.1149	105.3
Hypholomine B _MS_	0.0714	0.0344	0.1077	105.1
		0.0688	0.1433	104.4
		0.1032	0.1797	105.0

### Application of the method

The samples of *Phellinus igniarius* from Zhejiang Qingzheng Biotechnology Co. Ltd. which S10 and S11 are from the same source have been proved that they can increase the biosynthesis and secretion of bile acids to play the hypolipidemic activity in our previous research. Samples from other main productive places in china were collected. All samples were authorized with Prof. Chen Xilin of Zhejiang Chinese Medical University. Inoscavin A and Hypholomine B in twelve *Phellinus igniarius* samples were determined with UPLC-PAD-MS method. The results were listed in Table 5. The content of these compounds varied significantly in *Phellinus igniarius* samples growing in different locations. For instance, sample S9 had the highest contents of Inoscavin A and Hypholomine B, Sample S10 and S11 had a higher contents level as well. The amount of the target compounds identified by the two methods was in close agreement. Real sample data demonstrated that UPLC-PAD-MS was suitable for the analysis of these active components in *Phellinus igniarius* samples for the aim of quality control, the method could be adopted to investigate the influence of content on the effect as well. Further research should be carried out.

**Table 5:** Content of Inoscavin A and Hypholomine B in different *Phellinus igniarius* samples.

Sample ID	Harvest time	Place origins	Inoscavin A (g kg^-1^)^a^		Hypholomine B (g kg^-1^)	
			PAD	MS	PAD	MS
S1	June, 2015	Hangzhou	0.93 ±0.03	0.98±0.03	1.56 ±0.03	1.60±0.03
S2	July, 2015	Baishan	0.80 ±0.02	0.82±0.02	1.35 ±0.03	1.38±0.03
S3	May, 2015	Yuxi	1.03 ±0.05	1.10±0.06	1.18 ±0.04	1.23±0.04
S4	May, 2015	Shaotong	0.66 ±0.02	0.71±0.02	0.54 ±0.01	0.56±0.02
S5	July, 2015	Yanbian	0.80±0.02	0.83±0.01	1.38±0.02	1.43±0.03
S6	May, 2015	Lijiang	0.60±0.02	0.62±0.02	0.37±0.03	0.39±0.02
S7	May, 2015	Xishui	0.32±0.01	0.36±0.02	ND.^b^	ND.
S8	May, 2015	Kunming	ND.	ND.	0.32±0.03	0.35±0.02
S9	June, 2015	Bozhou	1.32±0.03	1.34±0.03	3.11±0.09	3.21±0.08
S10	June, 2015	Hangzhou	0.96±0.01	1.02±0.02	2.32±0.01	2.37±0.03
S11	June, 2015	Hangzhou	1.19±0.02	1.24±0.01	2.18±0.01	2.21±0.03
S12	June, 2015	Hangzhou	0.47±0.01	0.53±0.01	0.68±0.02	0.71±0.02

^a.^ Content = mean ± S.D. (n = 3).

^b^ Not detected.

### Comparison of UPLC and HPLC

**Fig 5:**
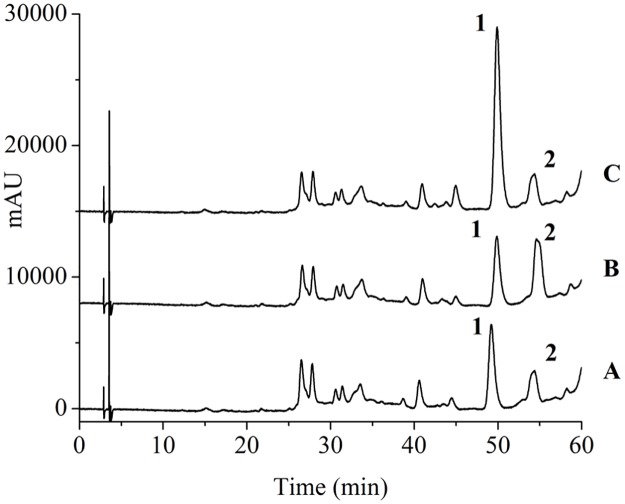
HPLC-UV chromatogram. of (A) blank sample; (B) sample spiked with Inoscavin A (20 mg L^-1^); (C) sample spiked with Hypholomine B (90 mg L^-1^). Hypholomine B (1), Inoscavin A (2).

The performance of the UPLC-PAD-MS method was compared with that of the HPLC-UV method, as shown in Fig 5. Good separation was achieved after screening a series of mobile phase and gradient profiles. The LODs of Inoscavin A and Hypholomine B using HPLC were 1.25 and 5.75 mg L^-1^, respectively. The UPLC-PAD-MS method appeared more sensitive than the HPLC-UV method.

Moreover, Inoscavin A and Hypholomine B were not well separated using the reported HPLC method [14], with the running time about 14min. Because the main structure of these two composition were same, their chromatographic behavior were similar, only when the initial ratio of organic solvent was low, can a necessary separation efficiency be gotten. As the result, the HPLC method needed a long analysis time, 60 min, whereas the UPLC-PAD-MS method required only 10 min. The combination of the shortened running time and a lower flow rate (0.3 mL min^-1^ vs 1.0 mL min^-1^) reduced solvent consumption from 60 mL to 3 mL per analysis. According to the Van Deemter equations, the efficiency of chromatographic performance was proportional to particle size decrease 23. In this study, the UPLC analytical column was packed with 1.7 μm particles while the conventional HPLC system contained 5 μm particle packed analytical columns.

## Conclusions

A rapid method for the simultaneous quantification of Inoscavin A and Hypholomine B in *Phellinus igniarius* samples was developed and evaluated. Quantitative analyses were performed using standard methods. HSCCC was more reliable for the preparation of Inoscavin A and Hypholomine B standards from *Phellinus igniarius* samples. Both the UPLC-PAD and UPLC-MS methods used in this work were well suited for the rapid routine quantification of these two compounds. Chromatographic separation permitted an analysis of the compounds in less than 10 min. A comparison was made between UPLC-PAD-MS and HPLC-UV in quantification analyses. UPLC-PAD-MS method showed many advantages including reduced analysis time, less solvent consumption and increased sensitivity.

## Supporting Information

S1 Fig HPLC of Hypholomine B. The HPLC of Hypholomine B.
(TIF)

S2 Fig HPLC of Inoscavin A. The HPLC of Inoscavin A.
(TIF)

S3 Fig MS spectrum 1 for Hypholomine B. The Molecular ion peak of Hypholomine B.
(TIF)

S4 Fig MS spectrum 2 for Hypholomine B. Analysis of Hypholomine B mass spectrum.
(TIF)

S5 Fig MS spectrum 1 for Inoscavin A. The Molecular ion peak of Inoscavin A.
(TIF)

S6 Fig MS spectrum 2 for Inoscavin A. Analysis of Inoscavin A mass spectrum.
(TIF)

S7 Fig ^1^H NMR 1 for Hypholomine B. The ^1^H NMR spectrum of Hypholomine B.
(TIF)

S8 Fig ^1^H NMR 1 for Inoscavin A. The ^1^H NMR spectrum of Inoscavin A.
(TIF)

S1 File Data analysis of ^1^H NMR. Data of ^1^H NMR of Hypholomine B and Inoscavin A.
(DOC)
