# Supplementary figures and images for: Simultaneous Quantification of Antioxidant Compounds in Phellinus igniarius Using Ultra Performance Liquid Chromatography-Photodiode Array Detection-Electrospray Ionization Tandem Mass Spectrometry
Source: PLoS One. 2016 Sep 30;11(9):e0163797. doi: 10.1371/journal.pone.0163797 (PMC5045194; doi:10.1371/journal.pone.0163797)

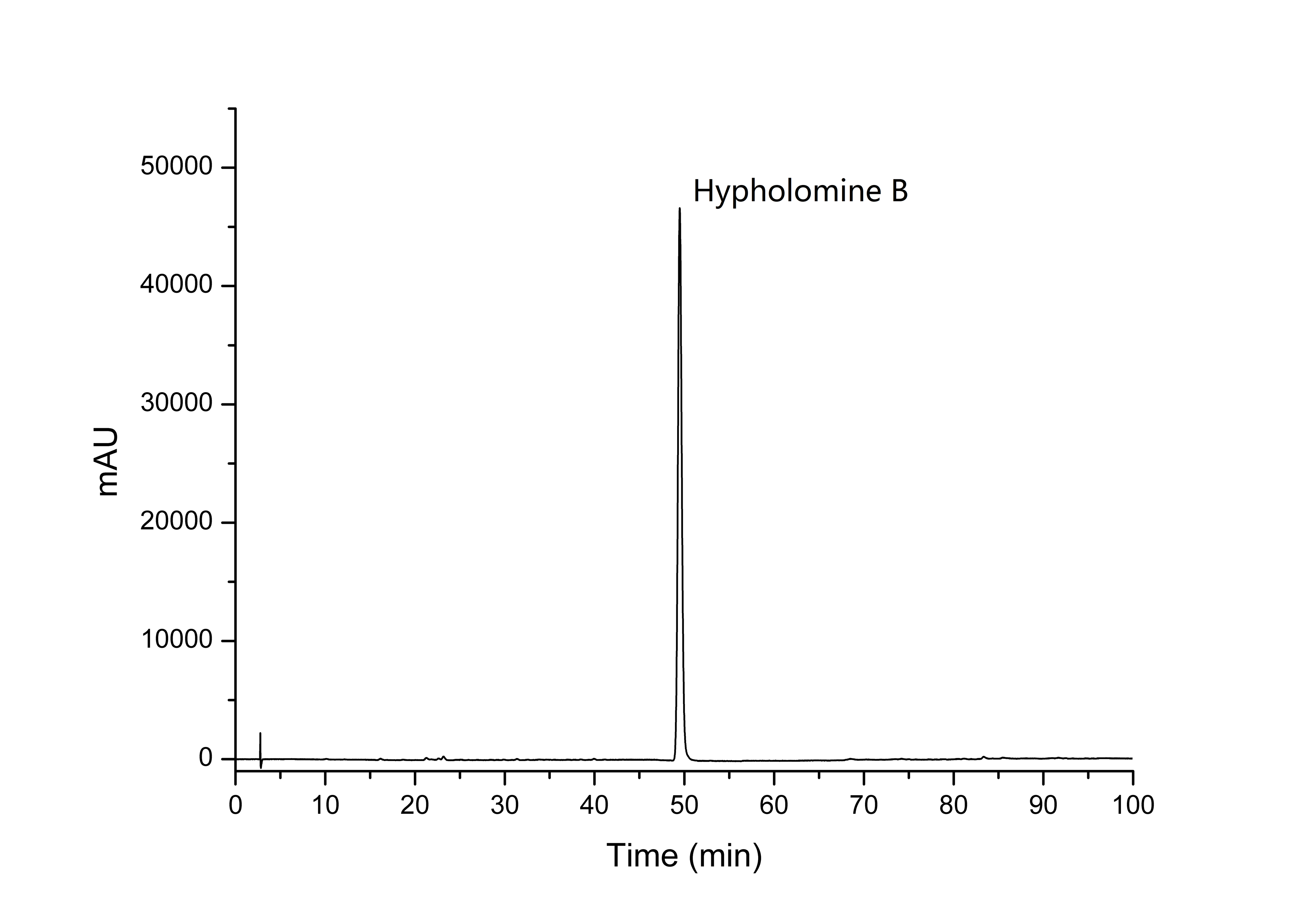

Supplement: S1 Fig — The HPLC of Hypholomine B. (TIF) [file pone.0163797.s001.tif]

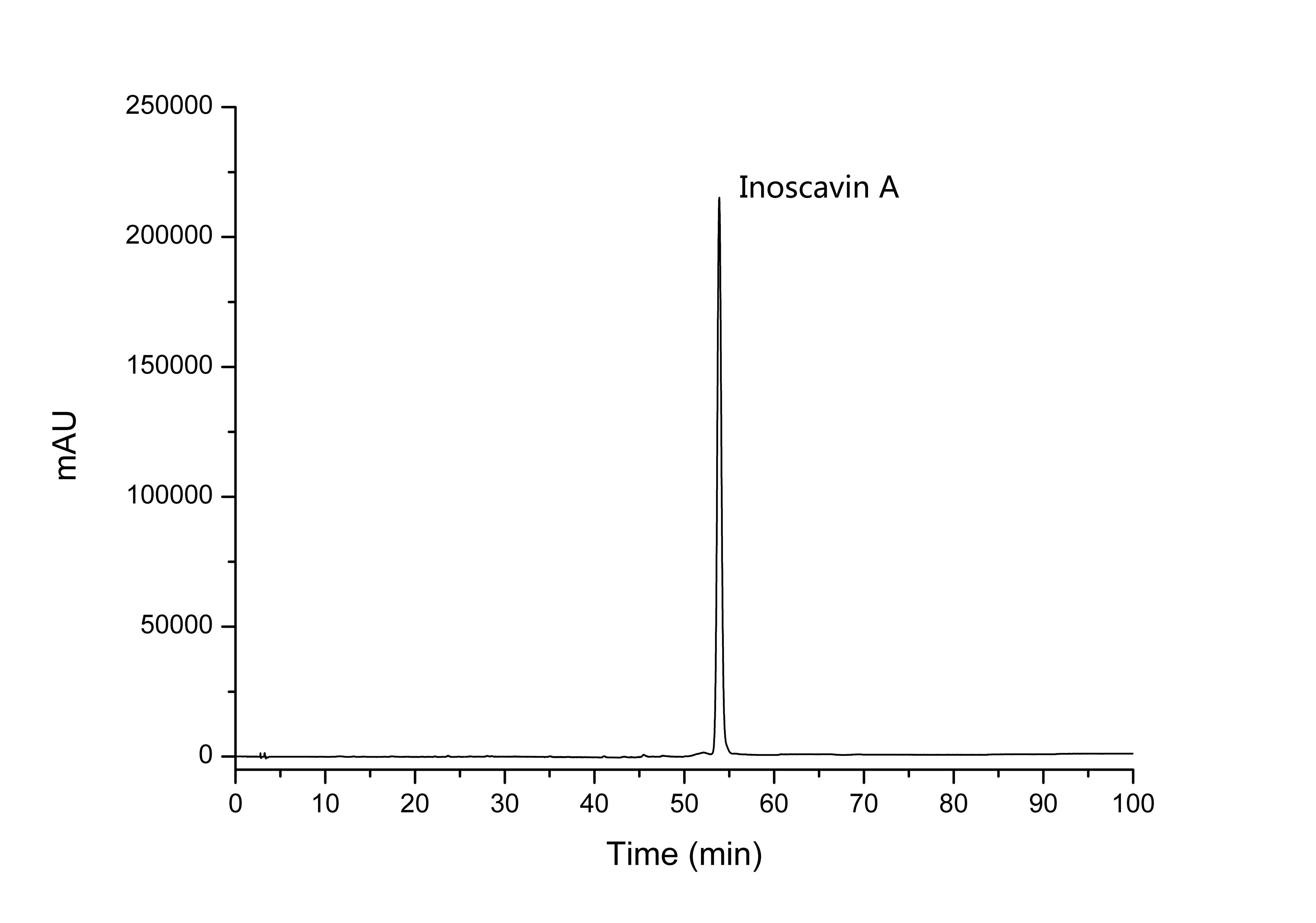

Supplement: S2 Fig — The HPLC of Inoscavin A. (TIF) [file pone.0163797.s002.tif]

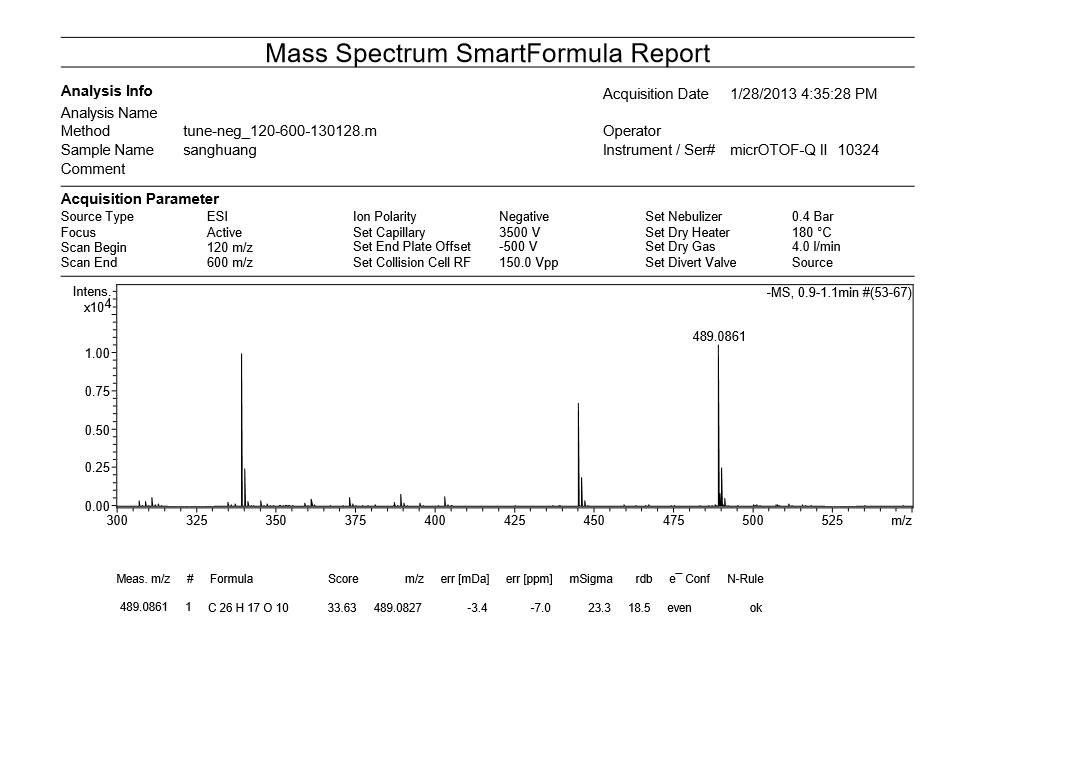

Supplement: S3 Fig — The Molecular ion peak of Hypholomine B. (TIF) [file pone.0163797.s003.tif]

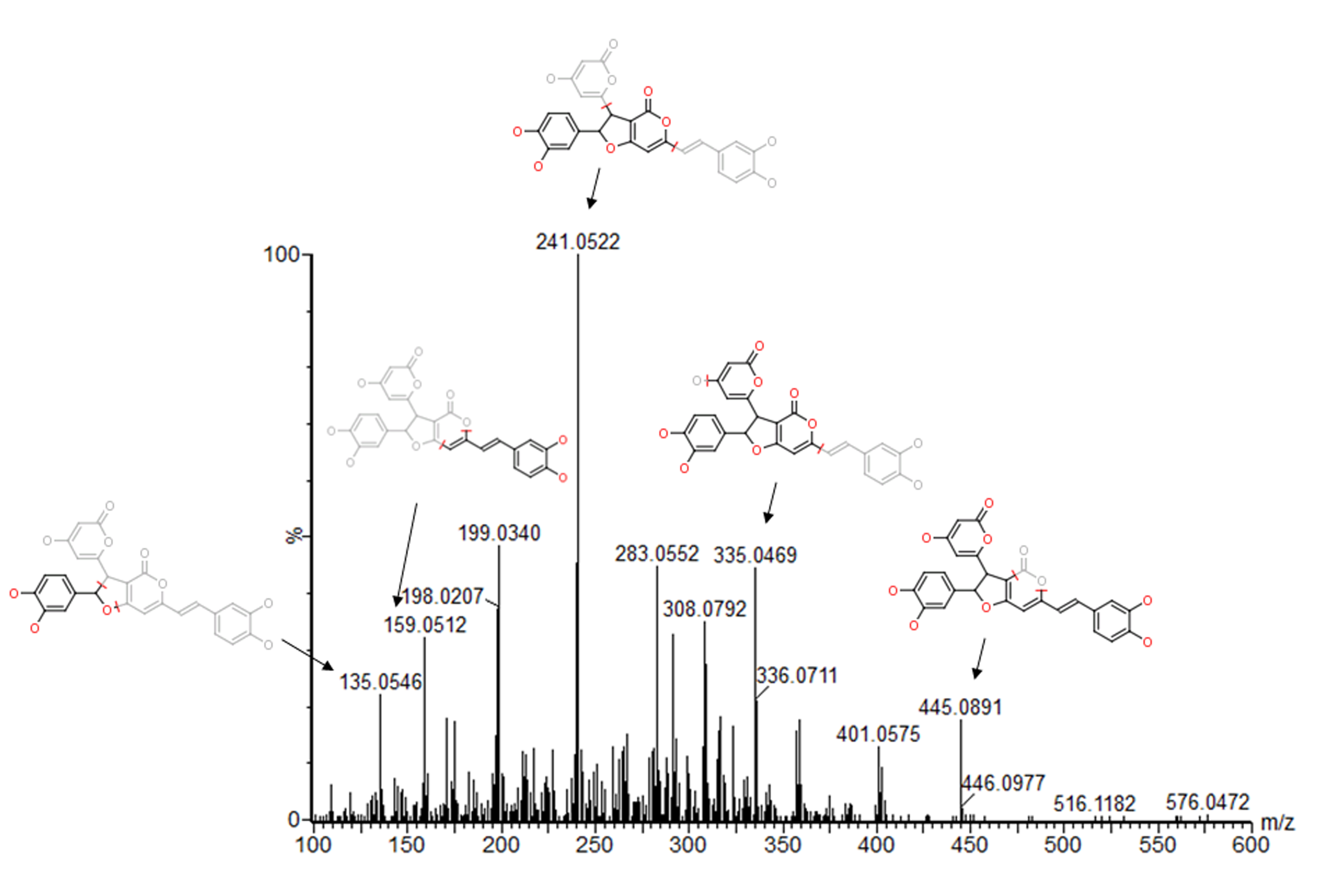

Supplement: S4 Fig — Analysis of Hypholomine B mass spectrum. (TIF) [file pone.0163797.s004.tif]

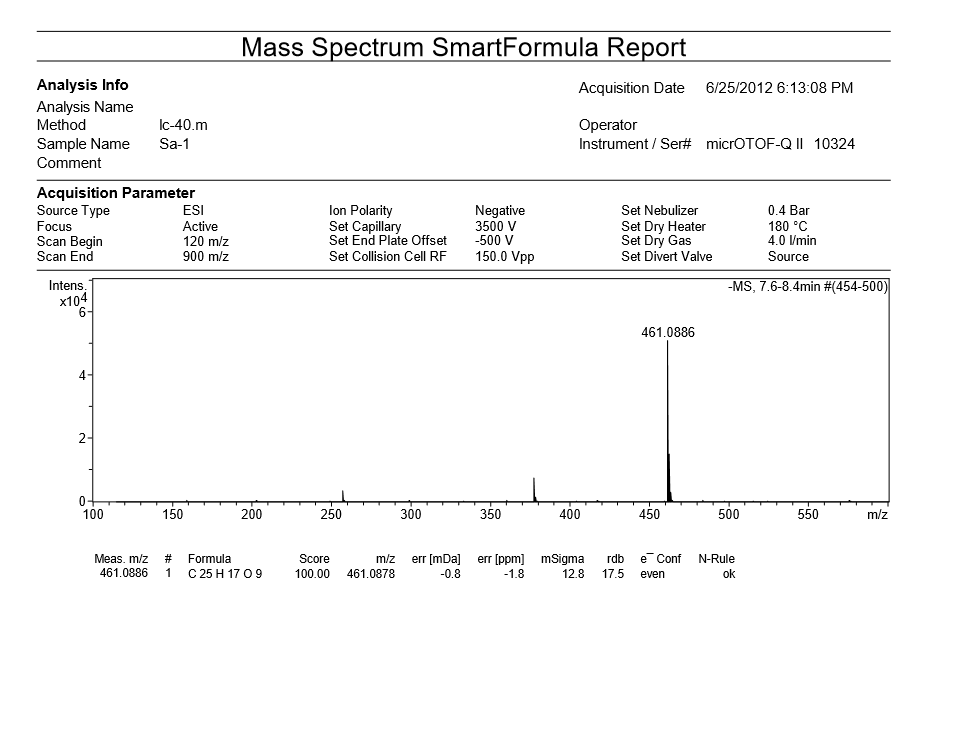

Supplement: S5 Fig — The Molecular ion peak of Inoscavin A. (TIF) [file pone.0163797.s005.tif]

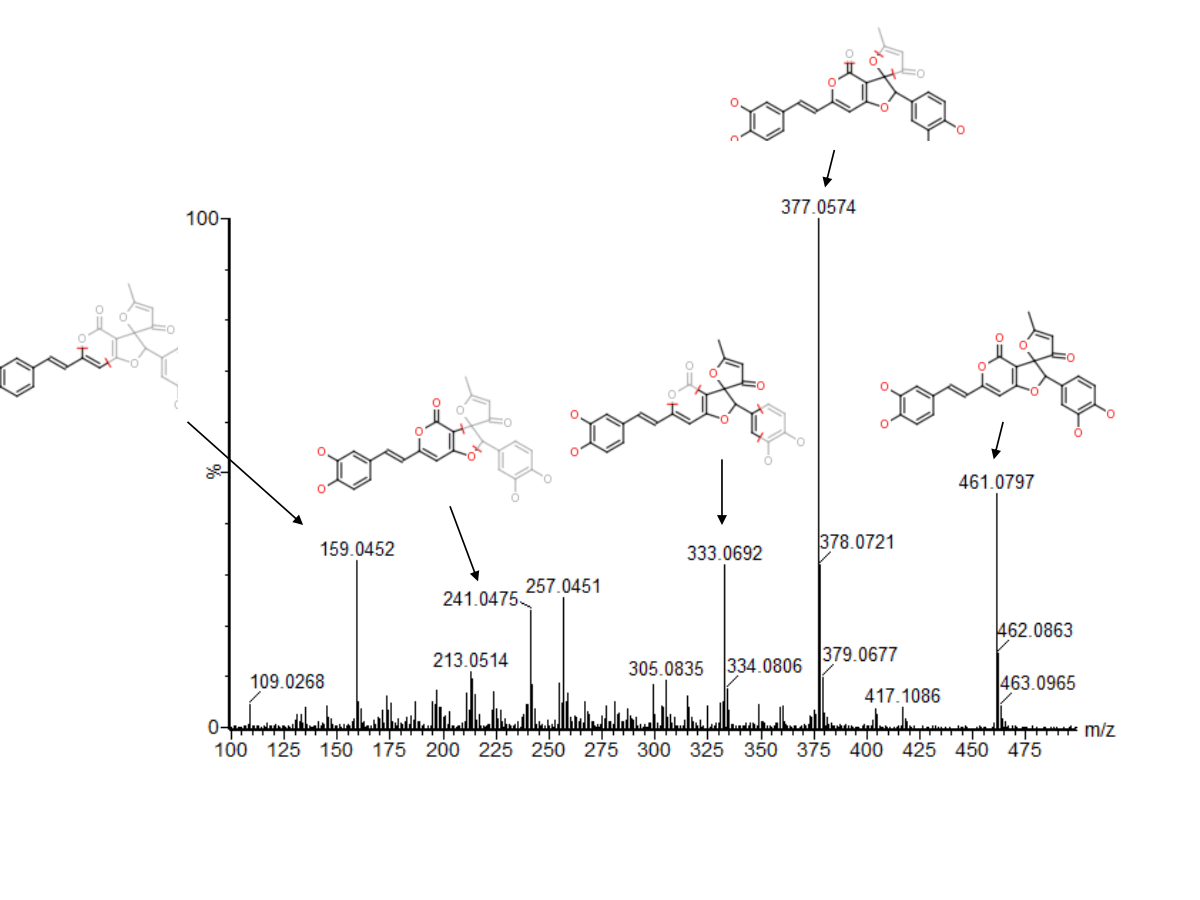

Supplement: S6 Fig — Analysis of Inoscavin A mass spectrum. (TIF) [file pone.0163797.s006.tif]

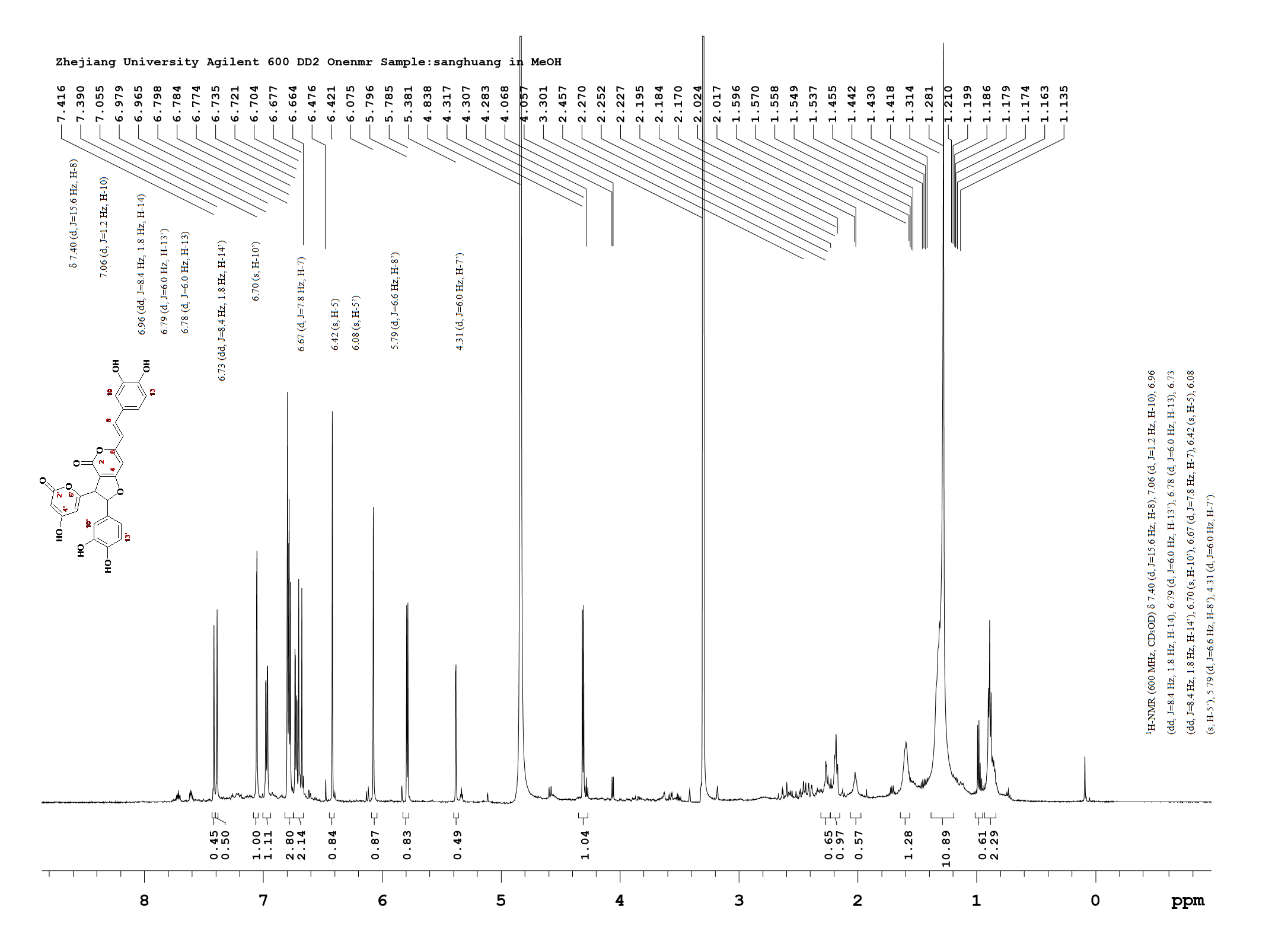

Supplement: S7 Fig — The 1H NMR spectrum of Hypholomine B. (TIF) [file pone.0163797.s007.tif]

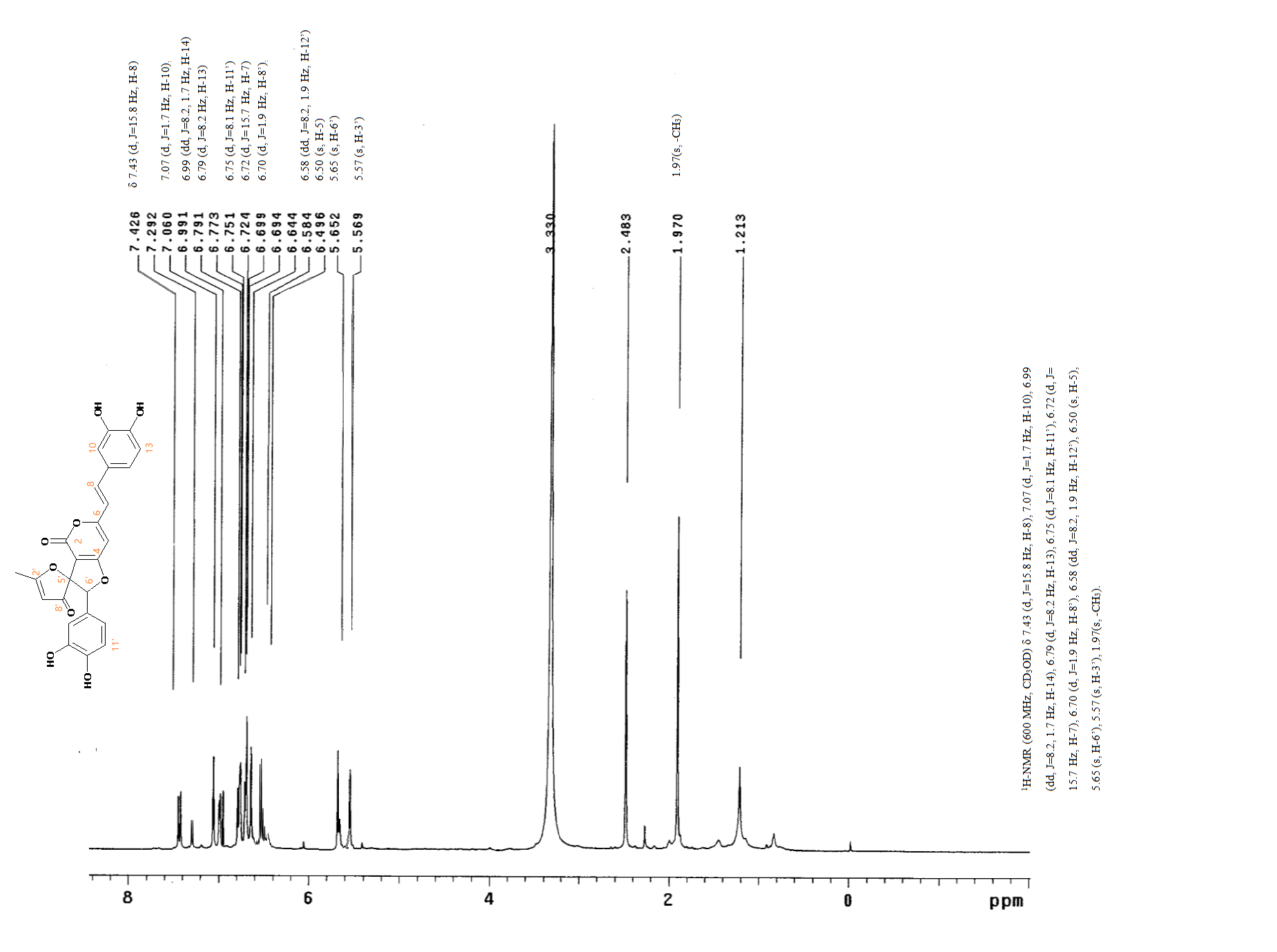

Supplement: S8 Fig — The 1H NMR spectrum of Inoscavin A. (TIF) [file pone.0163797.s008.tif]
