# Supplementary material for: Simultaneous Quantification of Antioxidant Compounds in Phellinus igniarius Using Ultra Performance Liquid Chromatography-Photodiode Array Detection-Electrospray Ionization Tandem Mass Spectrometry
Source: PLoS One. 2016 Sep 30;11(9):e0163797. doi: 10.1371/journal.pone.0163797 (PMC5045194; doi:10.1371/journal.pone.0163797)
Supplement: S1 File — Data of 1H NMR of Hypholomine B and Inoscavin A. (DOC) [file pone.0163797.s009.doc]

Hypholomine B

C_26_H_18_O_10_

^1^H-NMR (600 MHz, CD_3_OD) δ 7.40 (d, J=15.6 Hz, H-8), 7.06 (d, J=1.2 Hz, H-10), 6.96 (dd, J=8.4 Hz, 1.8 Hz, H-14), 6.79 (d, J=6.0 Hz, H-13’), 6.78 (d, J=6.0 Hz, H-13), 6.73 (dd, J=8.4 Hz, 1.8 Hz, H-14’), 6.70 (s, H-10’), 6.67 (d, J=7.8 Hz, H-7), 6.42 (s, H-5), 6.08 (s, H-5’), 5.79 (d, J=6.6 Hz, H-8’), 4.31 (d, J=6.0 Hz, H-7’).

Inoscavin A

C_25_H_18_O_9_

^1^H-NMR (600 MHz, CD_3_OD) δ 7.43 (d, J=15.8 Hz, H-8), 7.07 (d, J=1.7 Hz, H-10), 6.99 (dd, J=8.2, 1.7 Hz, H-14), 6.79 (d, J=8.2 Hz, H-13), 6.75 (d, J=8.1 Hz, H-11’), 6.72 (d, J= 15.7 Hz, H-7), 6.70 (d, J=1.9 Hz, H-8’), 6.58 (dd, J=8.2, 1.9 Hz, H-12’), 6.50 (s, H-5), 5.65 (s, H-6’), 5.57 (s, H-3’), 1.97(s, -CH_3_).
